# Use of Nitric Oxide Donor-Loaded Microbubble Destruction by Ultrasound in Thrombus Treatment

**DOI:** 10.3390/molecules27217218

**Published:** 2022-10-25

**Authors:** Ricardo Corro, Carlos Franco Urquijo, Oscar Aguila, Elisa Villa, Jesus Santana, Amelia Rios, Bruno Escalante

**Affiliations:** Cinvestav Monterrey, Centro de Investigación y de Estudios Avanzados del IPN, Apodaca 66600, Mexico

**Keywords:** thrombolysis, nitric oxide donors, microbubbles

## Abstract

In the presence of a vascular thrombus, the recovery of blood flow and vascular recanalization are very important to prevent tissue damage. An alternative procedure to thrombolysis is required for patients who are unable to receive surgery or thrombolytic drugs due to other physical conditions. Recently, the performance of thrombolysis combined with microbubbles has become an attractive and effective therapeutic procedure. Indeed, in a recent study, we demonstrated that, upon exposure to ultrasound, liposomes loaded with nitric oxide release agonists conjugated to microbubbles; therefore, there is potential to release the agonist in a controlled manner into specific tissues. This means that the effect of the agonist is potentiated, decreasing interactions with other tissues, and reducing the dose required to induce nitric-oxide-dependent vasodilation. In the present study, we hypothesized that a liposome microbubble delivery system can be used as a hydrophilic agonist carrier for the nitric oxide donor spermine NONOate, to elicit femoral vasodilation and clot degradation. Therefore, we used spermine-NONOate-loaded microbubbles to evaluate the effect of ultrasound-mediated microbubble disruption (UMMD) on thromboembolic femoral artery recanalization. We prepared spermine NONOate-loaded microbubbles and tested their effect on ex vivo preparations, hypothesizing that ultrasound-induced microbubble disruption is associated with the vasorelaxation of aortic rings. Thrombolysis was demonstrated in aorta blood-flow recovery after disruption by spermine NONOate-loaded microbubbles via ultrasound application in the region where the thrombus is located. Our study provides an option for the clinical translation of NO donors to therapeutic applications.

## 1. Introduction

The vascular endothelium is an important regulator of cardiovascular function. Endothelial dysfunction has been associated with a wide range of cardiovascular diseases, as well as pathological conditions, e.g., vasoconstriction, thrombosis, and an endothelial inflammatory state. Venous and arterial thromboses are important mechanisms for cardiovascular pathology. Arterial thrombosis is the leading pathophysiological condition in the development of myocardial infarction and is a leading cause of mortality [1]. In the presence of a vascular thrombus, the recovery of blood flow and vascular recanalization are very important in the prevention of heart and brain damage. Although the use of surgical or pharmacological thrombolysis has decreased the number of fatal events, an alternative procedure is required for patients who are unable to receive surgery or thrombolytic drugs due to physical conditions. Recently, thrombolysis via ultrasound (US) combined with microbubbles (MBs) has become an attractive and effective therapeutic procedure [2].

Several modulators of endothelial function offer potential therapeutic solutions [3]. However, systemic vascular treatment requires high doses of drugs and results in non-specific distribution to undesired target tissue or organs, which, in turn, increases side effects. In addition, nitric oxide (NO) modulators have a short half-life. The use of novel NO donors for cardiovascular pathologies has numerous advantages, such as decreasing systemic side effects and increasing therapeutic potential, but they are still not specific to damaged tissues. Amine-based diazennium diolates have been suggested as excellent NO donors. Indeed, spermine NONOate has been described as having as great an effect as NO-dependent proangiogenic factor, compared to other NONOates [4]. Additionally, spermine NONOate is also considered the most potent inhibitor of thrombus formation through platelet aggregation inhibition [5]. Therefore, in order to decrease side effects and increase therapeutic efficacy, the administered dose must be reduced, and the delivery of drugs must become more targeted to the specific circulatory bed. In order to explore this problem, several drug delivery strategies to protect the drug against degradation and optimize drug release, specifically at the site of action, have been described previously [6,7]. In a recent study, we demonstrated that, upon exposure to US, liposomes loaded with nitric oxide release agonists conjugated to microbubbles, potentially enabling the release of the agonist in a controlled manner into specific tissues. This potentiates the effect of the agonist, thereby decreasing the interactions with other tissues and reducing the dose required to induce nitric-oxide-dependent vasodilation [8]. Recently, it has been demonstrated that microthrombi dissolution in the hind limb muscle of rats can be achieved using ultrasound microbubble destruction; the effect of this treatment was associated with increased concentrations of nitric oxide in the muscle [9]. The same authors have also shown that microbubbles, when conjugated with sodium nitroprusiate, produced increased vascular blood flow in association with radical oxygen species production [10]. Liposomes have also been used as drug delivery vehicles; however, their efficiency has been compromised by the inefficient intracellular delivery of the encapsulated payload. To improve liposomes as drug-delivery molecules, the combination of the high drug-loading capacity of liposomes with the echogenic properties of microbubbles has suggested a hybrid system of liposomes for controlled drug release.

In the present study, we hypothesized that a liposome microbubble delivery system could be used as a hydrophilic agonist carrier for the nitric oxide donor, spermine NONOate, to elicit femoral vasodilation and clot degradation. Thus, our aim was to demonstrate in vivo thrombus resolution by spermine NONOate, an effect that can be potentiated when spermine NONOate is administered by US liposome-microbubble disruption. We prepared spermine NONOate-loaded liposomes attached to microbubbles using the non-covalent avidin–biotin conjugation. The triggered agonist—delivery by the UMMD effect on intra-arterial femoral clot formation—was assessed using a Doppler blood flow measurement.

## 2. Results

### 2.1. Characterization of the Spermine NONOate–Liposome–Microbubble Conjugate

The size distribution of empty microbubbles ranged from 0.2 to 5.4 μm, with a *Dm* of 1.1 ± 0.1 μm and a PDI of 56.2%. The size distribution of the spermine-NONOate-loaded liposome microbubble conjugate ranged from 0.2 to 5.1 μm, with a DM of 1.5 ± 0.2 μm and a PDI of 85%.

**Figure 1:**
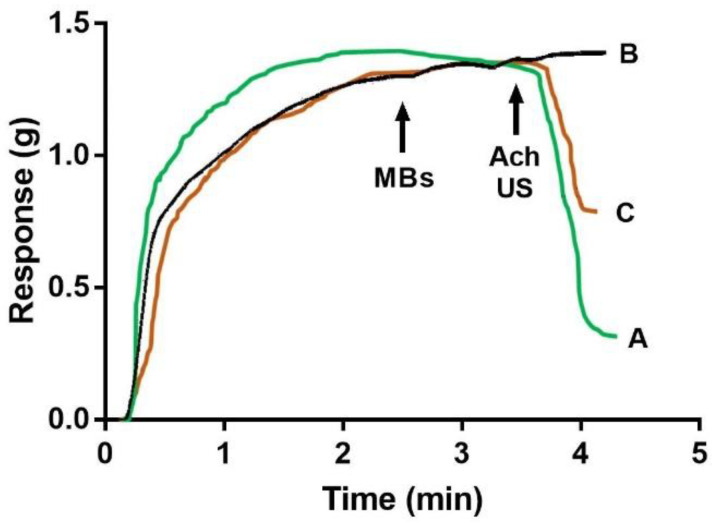
The Ultrasound-mediated microbubbles disruption1- UMMD effect of spermine-NONOate-loaded microbubble conjugates on vascular tone. The vascular response of rat aortic rings was evaluated in an isolated organ bath. Aortic rings were pre-contracted with phenylephrine before the addition of free ACh 1 × 10^−5^ M (A), empty microbubbles, or (B) spermine-NONOate-loaded microbubble conjugates. (C) Black arrows indicate whether microbubbles, acetylcholine, or US were applied to the aortic ring. Each trace represents one experiment of 4 aortic rings from 5 animals for each condition.

Spermine NONOate in vitro functional characterization—Spontaneous spermine NONOate release from liposomes was evaluated by testing its effect on vascular tension; after aortic ring incubation with liposomes without cholesterol, spontaneously reduced vascular tension was observed, whereas aortic ring incubation with liposomes with cholesterol did not change the vascular tension over the 1 h of experiment duration. Thus, the following experiments were performed using liposomes containing cholesterol. The pre-contracted aortic rings with spermine NONOate–liposome–microbubble (1 × 10^8^ microbubbles) conjugates were incubated for 5 min and then exposed to US, which led to a reduction in the phenylephrine-dependent precontracted vascular tone (Figure 1). The comparison of ACh-dependent relaxation, expressed as a percentage of the maximal relaxation response elicited by 1 × 10^−5^ M acetylcholine (see Figure 1), was 68 ± 4.35%, whereas, for aortic rings exposed to US and spermine, the NONOate–liposome–microbubble conjugate was 47.56 ± 3.23%. Furthermore, the absence of US stimulus to phenylephrine precontracted rings incubated with non-loaded liposome–microbubble (1 × 10^7^ microbubbles) resulted in no changes in the vascular tone (Figure 1). 

### 2.2. Spermine NONOate Vascular Relaxation Dose Response

**Figure 2:**
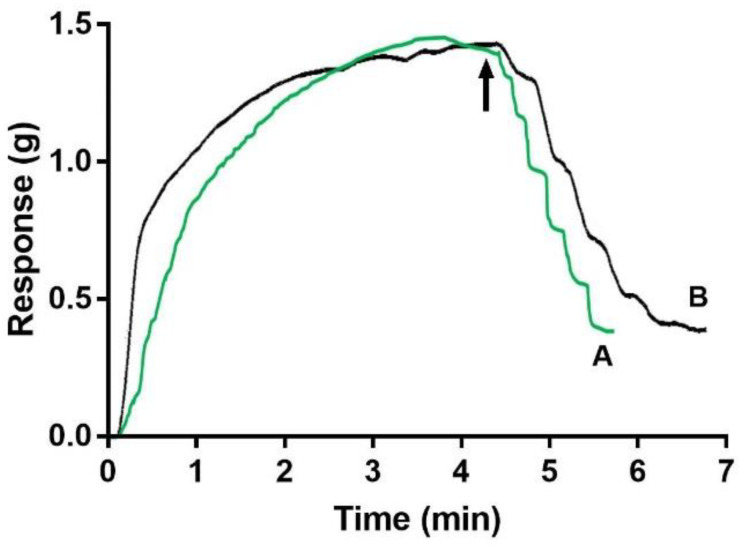
Dose-response curves for free spermine NONOate or spermine-NONOate-loaded microbubble conjugates after UMMD. The vascular response of rat aortic rings was evaluated after (A) an increasing concentration of free spermine NONOate or (B) an increasing number of spermine-NONOate-loaded microbubbles. Microbubble conjugates were added to the organ bath and then exposed to ultrasound (black arrow). Each trace represents one experiment with 4 aortic rings from 4 animals for each condition.

Spermine NONOate–liposome–microbubble conjugate dose-response curves (2.38 × 10^7^ to 50 × 10^7^ conjugates/mL) were obtained for the aortic rings by adding the conjugate into the organ bath, increasing the number of conjugates associated with the number of microbubbles, and then exposing them to US. Figure 2 shows the typical traces of a relaxation response curve that is dependent upon the increasing number of spermine NONOate liposome microbubble conjugates in the phenylephrine-pre-contracted aortic rings. 

**Figure 3:**
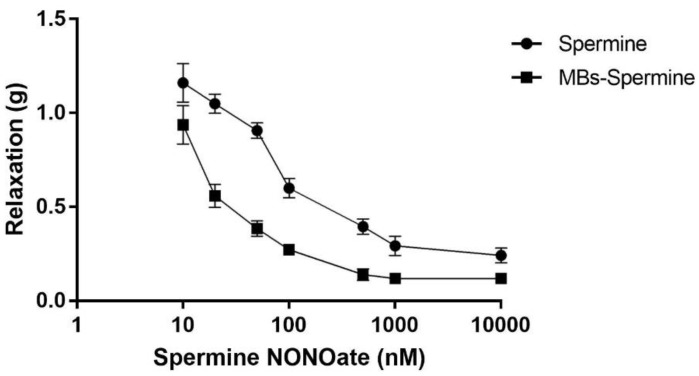
A comparison of dose-response curves for ultrasound-released spermine NONOate with free spermine NONOate. The vascular relaxation responses elicited by free spermine NONOate (10^−9^ to 10^−5^ M, circles) were plotted against spermine NONOate released by ultrasound from microbubble conjugates (squares). Each curve represents the mean ± SEM of 5 different experiments.

These curves were compared with the dose-response curves obtained after the addition of free spermine NONOate (Figure 3). Non-linear regression for each curve was used to calculate the ED50 values. Thus, the ED_50_ calculated for free spermine NONOate was 3.4 × 10^−7^ ± 3.8 × 10^−8^ M, while the ED_50_ for the spermine NONOate released by UMMD was 3.6 × 10^−8^ ± 2.8 × 10^−9^ M (*p* < 0.01).

### 2.3. Effect of Ultrasound Disruption of Spermine-NONOate-Loaded Microbubbles on Thrombolysis

**Figure 4:**
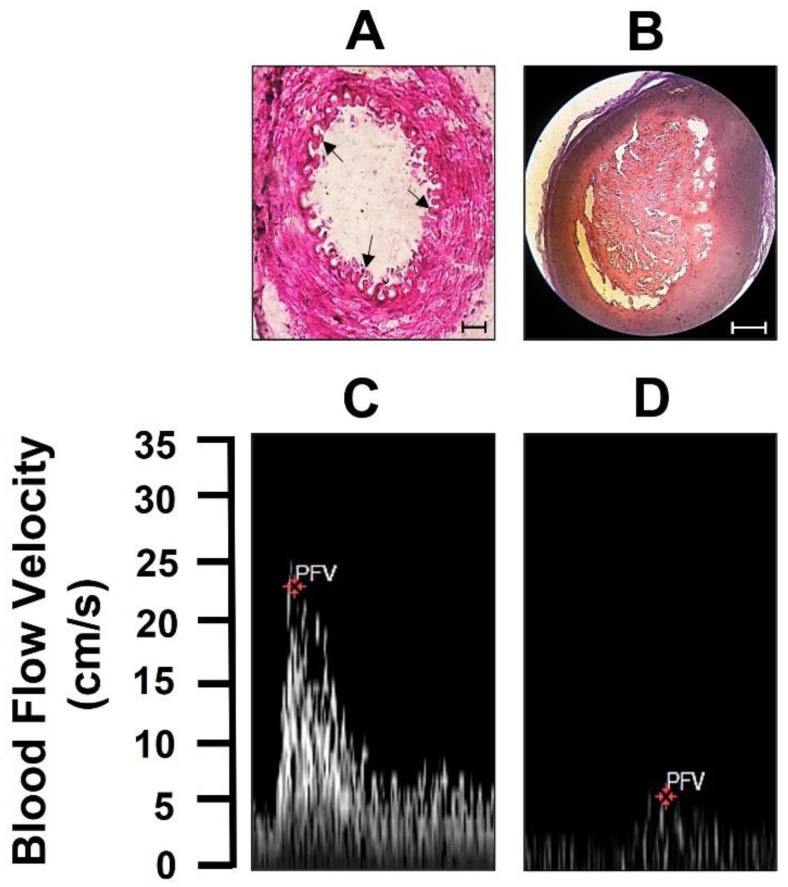
Femoral thrombus presence. (**A**) The upper panel shows pictures of the HE staining of the control femoral artery and (**B**) the presence of thrombus in the femoral artery (scale bar 100 μm). The lower panel shows femoral artery blood flow velocity (**C**) before and (**D**) after thrombus generation. Each trace or picture shows one experiment with 5 animals.

The effect of US stimulus on spermine-NONOate-loaded microbubbles and on thrombolysis was evaluated by femoral artery blood flow velocities in thromboembolic rats. The large thromboses in the femoral artery, confirmed by H&E staining (Figure 4, upper panel), show a control section of the femoral artery with no presence of thrombi in the lumen, whereas, in the femoral artery from thromboembolic rats, the lumen of the femoral artery was occluded by the presence of thrombi. Furthermore, the femoral artery blood flow velocity was clearly abolished compared to the blood flow velocity value before thrombus induction (Figure 4, lower panel). These data indicate the formation of an occlusive thrombus.

**Figure 5:**
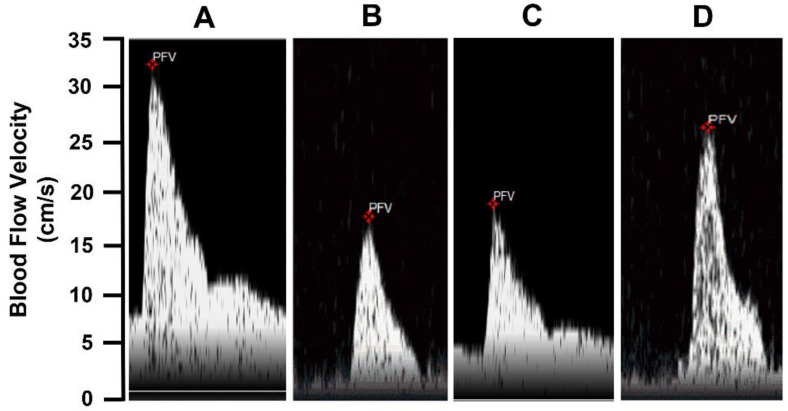
The thrombolytic effect of spermine NONOate+ UTMD. (**A**) Femoral blood flow velocity in control animals, (**B**) 24 h after thrombus formation without treatment, (**C**) 24 h after thrombus formation and free spermine NONOate treatment, and (**D**) 24 h after thrombus formation and spermine-NONOate-loaded microbubble and US stimulus treatments. Each trace or picture represents one experiment with 5 animals.

As shown in Figure 5, the blood flow velocity was lower in thromboembolic rats 24 h after thrombus formation (Figure 5B) compared to sham-operated rats (Figure 4A). Blood flow velocity values were also lower in thromboembolic rats treated with free spermine NONOate (Figure 5C). However, in thromboembolic rats treated with US and spermine-NONOate-loaded microbubbles (Figure 5D), blood flow velocity was higher compared to thromboembolic rats without treatment or free spermine-NONOate treatments.

**Figure 6:**
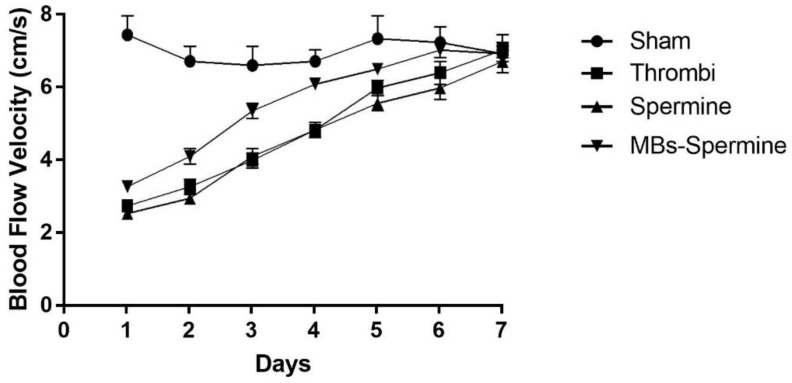
Time course femoral artery thrombus evolution. Each curve represents 7-day thrombus evolution in control animals (Sham), thrombus without treatment (Thrombi), free spermine NONOate treatment (spermine), and 24 spermine-NONOate-loaded microbubbles and US stimulus treatment (Mb spermine). Each curve represents the mean ± SEM of 5 experiments.

Furthermore, a time-course evaluation of the thrombus resolution demonstrated that artery recanalization was present in all groups after 7 days of thrombus formation. However, the improvement of the recanalization was faster in US spermine-NONOate-loaded microbubble-treated rats (Figure 6). 

## 3. Methods

### 3.1. Reagents

**Figure 7:**
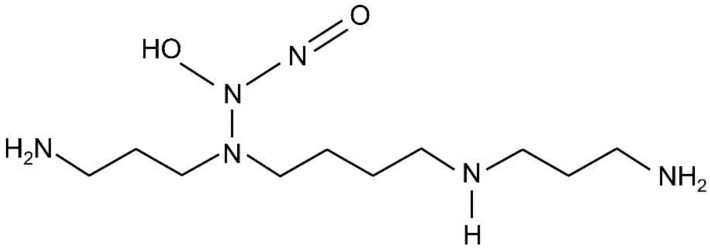
Spermine NONOate structure.

First, 1,2-Dipalmitoyl-sn-glycero-3-phosphocholine (DPPC),1,2-distearoyl-sn-glycero-3-phosphocholine (DSPC), avidin-FITC from egg white, (R)-(-)-phenylephrine hydrochloride (PHE), acetylcholine chloride (Ach), spermine NONOate (Figure 7), glycerol, propylene glycol, and chloroform were purchased from Sigma-Aldrich Corp. (St Louis, MO, USA). Then, 1,2-distearoyl-sn-glycero-3-phosphoethanolamine-N-[biotinyl(polyethyleneglycol)2000] (DSPE-PEG2000-Biotin), 1,2-distearoyl-sn-glycero-3-phosphoethanolamine-N-[amino(polyethyleneglycol)-2000] (DSPE-PEG2000), and cholesterol were purchased from Avanti Polar Lipids (Alabaster, AL, USA). Vybrant Dil was obtained from Molecular Probes (V-22885, Eugene, OR, USA). Perfluoropropane gas (C_3_F_8_) was purchased from Coastal Specialty Gas (Beaumont, TX, USA). The purity of all reagents was ≥99%.

### 3.2. Preparation of the Complex-Loaded Liposome Microbubbles

To create the spermine NONOate-loaded liposomes, two unilamellar liposomes (without and with cholesterol) loaded with spermine NONOate were prepared using a film hydration method [11]. Two different mixtures of phospholipids (DSPC: DSPE-PEG2000: DSPEPEG2000-Biotin (90:5:5)) or (DSPC: DSPE-PEG2000:DSPE-PEG2000-Biotin: Cholesterol (85:5:5:5)) were dissolved in chloroform. The organic solvent was evaporated under a steady stream of nitrogen. Any residual solvent was removed in a vacuum chamber for 3 h. The dry lipids were then hydrated with 20 mM HEPES buffer (pH 7.4) at 65 °C for 20 min to obtain a final lipid concentration of 2.0 mg/mL; at this point, 100 mM of spermine NONOate was added. The samples were then extruded (Avanti Polar Lipids, Alabaster, AL) through a polycarbonate membrane (200 nm pore size) to obtain spermine NONOate liposomes. To remove any excess non-encapsulated spermine NONOate, the liposomes were washed five times with a buffer solution (pH 7.4 phosphate-buffered saline (PBS): glycerol: propylene glycol (80:10:10 *v*/*v*)), by centrifugation for 1 h at 4 °C and 21,000× *g*. Finally, the liposomes were resuspended in 100 μL of the same solution. The final encapsulation efficiency was 3.7 ± 0.3%.

### 3.3. Biotinylated Microbubbles

The lipid monolayer shell mixture, consisting of (DPPC:DSPE-PEG2000: DSPE-PEG2000-Biotin (90:5:5)), was prepared as described above, and the resulting dried lipid film was hydrated in 20 mM HEPES buffer (pH 7.4) at a final lipid concentration of 5 mg/mL. Then, 0.5 mL of this solution was transferred to a 1.5 amber glass vial, which was sealed with a PTFE/silicone septum (Sigma-Aldrich), degassed, and refilled with C3F8 gas. Biotinylated microbubbles were prepared via a device (SDI, Effingham, IL, USA) at 4500 oscillations/s for 46 s. Biotinylated microbubbles were diluted in 0.5 mL of the HEPES buffer and centrifuged twice for 5 min at 470× *g* (Sorvall Legend Micro 21R Microcentrifuge, Thermo Fisher Scientific Waltham, MA, United States)

The solution containing microbubbles underneath the top cake was removed. The remaining cake was resuspended in 100 μL of HEPES buffer. The conjugation of biotinylated loaded liposomes with biotinylated microbubbles via avidin, in order to approach an avidin-saturated level—0.04 mg/mL of FITC-avidin—was added to 80 μL (6.64 × 10^8^) of the biotinylated microbubble suspension, mixed well, and incubated for 10 min at room temperature and shaken gently. The avidin-bound microbubbles were washed twice to remove excess free avidin and resuspended in 100 μL of HEPES buffer. The extruded liposomes (2.0 mg/mL) were added to this avidin-saturated suspension and incubated for 20 min at room temperature, then shaken gently. The microbubbles carrying the loaded liposomes were washed 3 times to remove free liposomes. Finally, the conjugates were resuspended in 100 μL of the HEPES buffer.

### 3.4. Characterization of Loaded Liposome Microbubble Conjugates

#### 3.4.1. Size Distribution

The size distribution and concentration of the avidin-bound microbubbles or the loaded liposome–microbubble conjugates were assessed via confocal microscopy (Leica TCSSP5, Germany) using a polydimethylsiloxane microfluidic chamber (1 × 1 × 0.02 mm), as previously described [12]. Briefly, avidin-bound microbubbles or loaded liposome–microbubble conjugates were diluted in a HEPES buffer (1:10) and placed into the microfluidic chamber to obtain confocal images (TCS SP5, Leica, Mannheim, Germany). Three independent preparations from each condition were evaluated. From each independent preparation, 3 chambers were evaluated, and 9 images were obtained from each chamber. Confocal images were analyzed using the ImageJ software (NIH, Bethesda, MA, USA). The polydispersity index (PDI) was determined from the standard deviation (*σ*) and the microbubble average diameter (*Dm*).

#### 3.4.2. Ultrasound Exposure Setup

A commercial mobile US device, which included a transducer probe and the related electronics to control the US parameters, was used in this study (Intelect Mobile Ultrasound 2776, Chattanooga Group, Hixson, TN, USA). A planar US transducer with an active area of 5 cm^2^ was mounted at the bottom of an acrylic water tank. The water-jacketed organ bath was positioned and aligned 2.5 cm above the transducer surface [6]. Water was used as a coupling medium between the transducer and the organ bath. Sinusoidal US waves of 1 MHz with an acoustic pressure of 0.43 MPa, a mechanical index of 0.2, a duty cycle of 10%, and a pulse-repetition frequency of 100 Hz were applied. The peak negative acoustic pressure generated at the center of the transducer was measured separately using a calibrated hydrophone (HNR-0500, Onda Corp., Sunnyvale, CA, USA). The US signal was monitored using a synchronized digital oscilloscope (USB-5132, National Instruments, Austin, TX, USA). All experiments were carried out at 37 °C.

#### 3.4.3. Ex Vivo Functional Assays

Rat aortic ring preparation—All the procedures conformed to the National Institutes of Health “Guide for the Care and Use of Laboratory Animals (1996)” and were approved by the Institutional Ethics Review Committee for Animal Experimentation of Cinvestav-IPN (approval no. 0170-15). Male Wistar rats (250–300 g of body weight) were fed a balanced diet and were provided water ad libitum. The rats were sacrificed by cervical dislocation; the thoracic aorta was carefully removed and placed directly into ice-cold Krebs–Henseleit bicarbonate solution (117.8 mM NaCl, 6.0 mM KCl, 1.6 mM CaCl_2_, 1.2 mM MgSO_4_, 1.2 mM KH_2_PO_4_, 24.2 mM NaHCO_3_). The liposome–microbubble conjugates were added; US was applied at 0 and 5 min of vascular tension recording. The vascular tension was recorded for an additional 10 min. To assess the effect on vascular response with spermine NONOate liposome–microbubble conjugate after UMMD, the aortic rings were pretreated with 1 × 10^−6^ M PHE until the maximal contraction response was reached. Then, aortic rings were incubated with spermine–liposome–microbubble conjugates (b, 4.7 × 10^7^ conjugates/mL) and exposed to US. In addition, pre-contracted aortic rings were incubated with 1 × 10^−5^ M ACh without exposure to US.

To assess the efficiency of US to disrupt microbubbles conjugated with loaded liposomes, aortic rings were incubated with 4.7 × 10^7^ conjugates/mL PHE-loaded liposome microbubble conjugates, then exposed to US. Vascular tension was recorded. After reaching the maximal vascular tension, aortic rings were consecutively exposed to US three more times at 1-minute intervals, and changes in vascular tension were recorded.

#### 3.4.4. Dose–Response Curve for Free Spermine NONOate or Spermine NONOate–Liposome–Microbubble Conjugates

Concentration-response curves after UMMD were obtained for rat aortic rings (*n* = 4 rats) via the cumulative addition of conjugate related to an increasing number of microbubbles in the organ bath. In the case of the spermine NONOate–liposome–microbubble conjugate, the aortic rings were precontracted with 1 × 10^−6^ M PHE. In addition, the concentration-response curves for free spermine NONOate (10^−9^ to 10^−5^ M) were also obtained in rat aortic rings (*n* = 5 rats) via the cumulative addition of the drug to the organ bath. The concentration was increased only after a maximal response to the previous concentration was attained. Aortic rings were pre-contracted with 1 × 10^−6^ M PHE. These dose-response curves were used as a bioassay to determine either spermine NONOate or PHE uptake into the liposomes. Based on the amount of spermine-NONOate-loaded liposomes conjugated to microbubbles (20 ± 1.5 μg/2.5 × 10^8^ conjugates), the concentration in each volume of liposome–microbubble conjugates was calculated, plotted as a dose-response curve, and compared with the free spermine NONOate dose-response curves. The ED_50_ for each drug was calculated from the dose-response curves using the GraphPad software, Prism (GraphPad Prism version 6.00 for Windows, GraphPad Software, San Diego, CA, USA).

### 3.5. Thrombus Formation

The left hind limb of adult male Wistar rats was anesthetized. Then, a lateral incision was made to expose the femoral artery, which was dissected from the crural arch to the popliteal bifurcation to isolate it from the surrounding tissue. A small piece of plastic was placed under the artery to avoid damaging the surrounding tissue. A 1 cm-long filter paper containing 10% FeCl_3_ was placed on the surface of the isolated artery for 5 min. After the removal of the filter paper, the artery was rinsed with 0.9% sterile saline, and then the incision was stitched closed. The thrombus was observed for 2 h before treatment to guarantee stability. A Doppler flow velocity system (Indus Instruments Tx Houston USA) was used to determine whether a completely occluded blood clot had formed and remained stable.

### 3.6. Femoral Artery Recanalization

The rats were randomly distributed into four groups, with five rats in each group: (1) the sham-operated group—surgery was performed, but thrombus formation was not induced. (2) The control group—a saline solution was injected into the tail vein at a speed of 5 mL/h for 1 min. (3) The spermine-NONOate-free group—spermine NONOate (0.2 mg/mL) was injected into the tail vein at a speed of 5 mL/h for 1 min. (4) The UTMD group—spermine-NONOate-loaded MB suspension (1.10 × 10^8^ MBs/mL) was injected into the tail vein at a speed of 5 mL/h for 1 min. All groups of rats simultaneously received US irradiation for 1 min by placing the US probe in the femoral muscle (sinusoidal US waves of 3 MHz, with an acoustic pressure of 0.43 MPa, a mechanical index of 0.2, a duty cycle of 10%, and a pulse-repetition frequency of 100 Hz were applied). After treatment, the femoral blood flow velocity was measured by placing the blood flow velocity probe in the femoral muscle distal to the thrombus localization. 

To confirm the existence of thrombi in vivo and the effect of thrombolysis, femoral artery specimens from the rats were embedded in paraffin and sliced (5 μm). The sliced samples were deparaffinized, stained with hematoxylin and eosin (H&E), and counterstained with hematoxylin.

### 3.7. Statistical Analysis

All data are expressed as mean ± standard error of the mean (SEM). The one-way analysis of variance (ANOVA) and Tukey’s test were used for multiple comparisons. Data were evaluated using GraphPad (GraphPad Software, San Diego, CA, USA).

## 4. Discussion

In the present study, we used spermine-NONOate-loaded liposomes and microbubbles to evaluate the effect of UMMD on thromboembolic femoral artery recanalization. We prepared spermine-NONOate-loaded microbubbles and demonstrated ex vivo that US-induced microbubble disruption was associated with the vasorelaxation of aortic rings. Thrombolysis was demonstrated in aorta blood flow recovery after spermine-NONOate-loaded microbubble disruption, by US application in the region of thrombus localization. We suggest that spermine NONOate is released from the microbubbles under a US stimulus at a nanomolar concentration. Indeed, the aortic ring vasorelaxation induced by free spermine NONOate was comparable to that induced by spermine-NONOate-loaded microbubbles and released by US. Moreover, spermine NONOate-loaded liposomes-microbubbles were stable and did not elicit changes in vascular tension when they were not stimulated with US. These data support the idea that the lipid shell from both liposomes and microbubbles must first be ruptured to enable the spermine NONOate to interact with the vascular tissue and induce a significant change in vascular tone. Drug release was achieved in the first 10 s after exposure to US, reaching the maximum effect in less than 1 min. No further vascular responses were elicited after three consecutive iterations of US stimuli. Thus, the total amount of released drug was available upon the first US stimulus and elicited the maximal biological effect. This result contrasts with the report of the several acoustic cycles required for the complete release of NO from microbubbles [13]. Furthermore, the dose-response curves for free spermine NONOate and US-exposed spermine NONOate–liposome microbubble conjugates were different. The curves of the released spermine NONOate showed displacement to the left, suggesting that the administration of these agonists in liposome–microbubble conjugates accompanied by US-mediated release might potentiate the pharmacological effect. These findings are in agreement with our previous studies [6]. Additionally, other authors have demonstrated the synergistic effect of US and microbubbles [14,15]. The potentiation of the pharmacological effect must be associated with the facilitation of the drug’s entry into the cell, due to transient pore formation on cell membranes during the destruction of the microbubbles [16]. A violent collapse of microbubbles can produce fluid micro-jets and shear stress, increasing the permeability of biological barriers [17]. Recently, we demonstrated that UMMD enhanced the uptake of caveolin, an important regulator of endothelial nitric oxide synthase (eNOS), in endothelial cells [18].

After demonstrating that spermine-NONOate-loaded microbubbles can be stimulated by US to elicit predictable vascular function, we tested the effect of this spermine-NONOate preparation in the resolution of femoral artery thrombus. Our data show increased femoral blood flow velocity, demonstrating that the treatment of the femoral thrombus with spermine-NONOate-loaded microbubbles produces better results than with the intravenous administration of free spermine NONOate. Femoral thrombus resolution requires 7 days’ evolution without treatment; a similar amount of time was required in rats treated with free spermine NONOate. However, treatment with US-released spermine-NONOate-loaded microbubbles required 4 days to resolve the thrombus. Furthermore, 1 day after the treatment was applied, the thrombus resolution was better than in the other groups. There are several mechanisms that may be associated with thrombolysis enhancement by the US disruption of spermine-NONOate-loaded microbubbles. First, it is important to mention that, with the application of US alone, the thrombus can still be dissolved, and also that recanalization is possible, regardless of re-occlusion. This is perhaps due to the fact that US can immediately change the fibrin structure, generating better penetration in the tissue plasminogen activator. The thrombus absorbs the ultrasound energy, which in turn, causes an increase in temperature, thereby accelerating the enzymatic hydrolysis of fibrin [19]. Data from other studies also indicate that US itself was capable of damaging the thrombus, primarily by distracting the fibrin matrix. This is very important for thrombolysis therapy because US, in damaging the matrix of the thrombus, can prevent the fiber proteins and platelets from re-assembling, thus reducing the chances of re-occlusion [20].

US has been proposed as an alternative for angiogenesis rescue [21]. Thus, it is possible that US stimulates nitric oxide release in the side of the thrombus, contributing to the increased femoral blood flow velocity observed in the control group (thrombus without treatment). Second, empty microbubbles stimulated by US have been described as generating two physical processes: (a) cavitation, when the microbubbles are under stable cavitation; (b) oscillation, where the oscillation of microbubbles results in microflow generation that can produce erosion of the thrombus surface [16]. Alternatively, it is possible that, during microbubble collapse, microflow jets are created, breaking the fibrin network. [17]. Third, the combination of US, microbubbles, and spermine NONOate release may be associated with an increased concentration of nitric oxide in the thrombus, resulting in improved thrombus resolution. This hypothesis is supported by a previous study showing that in the presence of nitric oxide synthesis inhibition, the effect of microbubbles and US on vascular blood flow perfusion is prevented [22]. A recent study using NO-MBs significantly improved the circulation time of NO in vivo. Moreover, the release of a therapeutic treatment via US-targeted microbubble destruction (UTMD) caused a significant decrease in the thrombus area and an increase in the recanalization rates and blood flow velocities, compared to the control and US groups [23]. In our data, we have demonstrated that spermine-NONOate-loaded microbubbles improved femoral artery recanalization, as shown by the increased femoral blood flow velocity 48 hours after thrombus formation. It is possible to speculate that US and MB stimuli in the thrombus area may stimulate the vascular endothelium, resulting in nitric oxide release. However, the nitric oxide concentration is not sufficiently high to contribute to the improvement of thrombus resolution; moreover, the short half-life of nitric oxide may result in a short-term effect of endothelium-dependent nitric oxide release. Thus, it may be feasible that the use of a more stable nitric oxide donor, such as spermine NONOate, will have a more potent and larger effect on thrombus resolution. Our data show that femoral artery blood flow velocity normalization, seen in rats treated with spermine-NONOate-loaded microbubbles stimulated by US, may have contributed to the activation of eNOS and increased the bioavailability of NO. However, further experiments should be performed to demonstrate this hypothesis.

In conclusion, spermine-NONOate-loaded microbubble disruption by US is effective in enhancing thrombolysis, while causing NO release. Spermine-NONOate-loaded MBs probably achieved large-vessel thrombolysis and protection against tissue ischemia. Our study provides an option for the clinical translation of NO donors to therapeutic applications. Therefore, clinical studies are necessary to demonstrate the advantages of the use of UMMD versus conventional methods.

## Data Availability

Not applicable.
